# Detection of Pathogenic Viruses, Pathogen Indicators, and Fecal-Source Markers within Tanker Water and Their Sources in the Kathmandu Valley, Nepal

**DOI:** 10.3390/pathogens8020081

**Published:** 2019-06-19

**Authors:** Bikash Malla, Rajani Ghaju Shrestha, Sarmila Tandukar, Dinesh Bhandari, Ocean Thakali, Jeevan B. Sherchand, Eiji Haramoto

**Affiliations:** 1 Interdisciplinary Center for River Basin Environment, University of Yamanashi, 4-3-11 Takeda, Kofu, Yamanashi 400-8511, Japan; mallabikash@hotmail.com; 2 Division of Sustainable Energy and Environmental Engineering, Osaka University, Suita, Osaka 565-0871, Japan; rajani_ghaju12@hotmail.com; 3 Department of Natural, Biotic and Social Environment Engineering, University of Yamanashi, 4-3-11 Takeda, Kofu, Yamanashi 400-8511, Japan; sar1234tan@gmail.com; 4 Institute of Medicine, Tribhuvan University Teaching Hospital, Kathmandu 1524, Nepal; me.dinesh43@gmail.com (D.B.); jeevanbsherchand@gmail.com (J.B.S.); 5 Environmental and Social System Science Course, University of Yamanashi, 4-3-11 Takeda, Kofu, Yamanashi 400-8511, Japan; othakali@gmail.com

**Keywords:** fecal-source marker, index virus, microbial contamination, pathogenic virus, tanker water

## Abstract

Tanker water is used extensively for drinking as well as domestic purposes in the Kathmandu Valley of Nepal. This study aimed to investigate water quality in terms of microbial contamination and determine sources of fecal pollution within these waters. Thirty-one samples from 17 tanker filling stations (TFSs) and 30 water tanker (WT) samples were collected during the dry and wet seasons of 2016. *Escherichia coli* was detected in 52% of the 31 TFS samples and even more frequently in WT samples. Of the six pathogenic viruses tested, enteroviruses, noroviruses of genogroup II (NoVs-GII), human adenoviruses (HAdVs), and group A rotaviruses were detected using quantitative PCR (qPCR) at 10, five, four, and two TFSs, respectively, whereas Aichi virus 1 and NoVs-GI were not detected at any sites. Index viruses, such as pepper mild mottle virus and tobacco mosaic virus, were detected using qPCR in 77% and 95% out of 22 samples, respectively, all of which were positive for at least one of the tested pathogenic viruses. At least one of the four human-associated markers tested (i.e., BacHum, HAdVs, and JC and BK polyomaviruses) was detected using qPCR in 39% of TFS samples. Ruminant-associated markers were detected at three stations, and pig- and chicken-associated markers were found at one station each of the suburbs. These findings indicate that water supplied by TFSs is generally of poor quality and should be improved, and proper management of WTs should be implemented.

## 1. Introduction

Kathmandu, the capital city of Nepal, faces a severe scarcity of water in terms of both quality and quantity [1,2,3,4]. Kathmandu Upatyaka Khanepani Limited (KUKL), the sole organization responsible for supplying piped water into the valley, can only supply 111 million liters per day (MLD) and 71 MLD in wet and dry seasons, respectively, while the actual demand approaches 377 MLD [4]. Therefore, to meet daily requirements for domestic water, households in the valley are compelled to employ alternative water sources [5]. Commonly used alternative water sources include groundwater (e.g., shallow dug and deep tube wells, and stone spouts), jar water, tanker water, and surface water sources, such as springs and rivers. Tanker water is a major component of the valley’s water market [6], as is so in other countries, such as Bangladesh, Indonesia, Pakistan, the Philippines, and Thailand [7]. Water tankers play an important role in transporting large volumes of water abstracted from ground and surface sources to communities and households lacking the infrastructure or that are deprived of water sources [6,8,9,10]. The sources of tanker water in the valley range from surface water to shallow or deep borings, whereas the treatment procedures usually applied by TFSs vary from aeration, sedimentation and filtration (generally by pressurized sand filters), to use of bleaching powders [6]. The number of tanker water consumers has been gradually increasing and has increased rapidly following the Gorkha Earthquake of 2015 [5]. Currently, 22% of households are using tanker water, of which 18%, 60%, 97%, and 95% use it for drinking, cooking, bathing, and laundry, respectively [5].

A previous study [11] reported the detection of fecal indicator bacteria and pathogens as well as ruminant fecal markers in tanker water supplied to a household. A recent study showed that 77% of tanker water samples collected in the valley exceeded the Nepal Drinking Water Quality Standard guideline for total coliform count [12]. Such findings have indicated possible public health risks associated with using tanker water.

Viruses such as pepper mild mottle virus (PMMoV) and tobacco mosaic virus (TMV) have been proposed as potential indicators of pathogenic viruses [13]. Pathogenic viruses, including Aichi virus 1 (AiV-1), human adenoviruses (HAdVs), enteroviruses (EVs), noroviruses of genogroups I and II (NoVs-GII), and group A rotaviruses (RVAs), have been studied to estimate the concentration of pathogenic viruses in various water sources [13,14]. However, data regarding tanker water are limited. Thus, there is a need to investigate microbial contamination and sources of fecal pollution in TFS samples and water distributed by WTs.

Prevention of potential disease outbreaks can be achieved by identifying sources of fecal contamination and formulating appropriate pollution mitigation strategies. Sources of fecal contamination can be identified by the application of a technique called microbial source tracking (MST), which accurately and reliably identifies the hosts responsible for fecal pollution [15,16]. Host-associated *Bacteroidales* assays—BacHum (human-associated) [17], BacR (ruminant-associated) [18], and Pig2Bac (pig-associated) [19] and mitochondrial DNA (mtDNA) markers (bovine-, dog-, and pig-associated) [20,21], as well as viral markers specific for humans (HAdVs) [22], JC and BK polyomaviruses (JCPyVs and BKPyVs) [23], chicken (chicken parvoviruses (ChkPVs) [24], and pig (porcine adenoviruses (PoAdVs) [25])—are commonly used for source tracking.

Based on this background, the current study aimed to assess the prevalence and abundance of pathogenic viruses and indicators of pathogens in order to identify sources of fecal contamination in TFSs and WT samples in the Kathmandu Valley.

## 2. Results

### 2.1. Detection of Fecal Indicator Bacteria and Index Viruses

Table 1 shows the positive ratios and concentration ranges of fecal indicator bacteria and index viruses (PMMoV and TMV) within water samples from TFSs and WTs. *Escherichia coli* and total coliforms were detected in 52% and 87% of 31 TFS samples, respectively, and were more frequent in WT samples. The mean concentration of *E. coli* in WT samples was 0.37 log greater than that in TFS samples, although the difference was not significant (independent *t*-test; *p* > 0.05). PMMoV and TMV were detected in 71% and 90% out of 31 TFS samples, respectively, whereas in WT samples, PMMoV and TMV were detected in 73% and 97% out of 30 samples, respectively. Of the 22 samples that were positive for at least one pathogenic virus, PMMoV and TMV were detected in 77% and 95% of samples, respectively. The *E. coli* concentrations were 0.0–4.0 and 0.0–3.5 log most probable number (MPN)/100 mL in TFSs and WT samples, respectively. Similarly, out of the two index viruses tested, TMV was detected with the highest concentration (6.3 log copies/L) in WT samples, whereas PMMoV was detected with the lowest concentration (1.7 log copies/L) in TFS samples. *E. coli* was detected in 44% (7/16) and 60% (9/15) of TFS samples during the dry and wet seasons, respectively, whereas it was detected in 65% (11/17) and 77% (10/13) of WT samples during the dry and wet seasons, respectively. Although the difference was not significant, the mean concentration of *E. coli* in WT samples during the wet season was 0.78 log greater than that within the dry season (independent *t*-test; *p* > 0.05).

**Figure 1:**
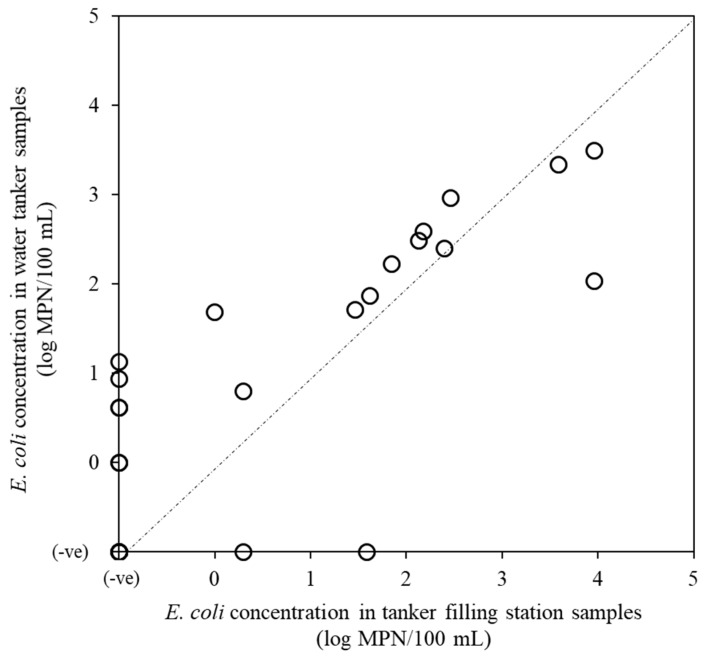
*E. coli* concentrations in tanker filling station and water tanker samples.

Figure 1 shows the *E. coli* concentration of water samples in the corresponding TFSs and WTs (27 pairs). In most cases, the *E. coli* concentration of WT samples was greater than that of corresponding TFS samples, although the mean concentrations did not differ significantly between WT (0.8 ± 1.6 log MPN/100 mL) and TFS samples (0.5 ± 1.8 log MPN/100 mL) (paired *t*-test, *p* > 0.05). Forty-six percent (6/13) of *E. coli*-negative TFS samples were positive for *E. coli* in the corresponding WT samples.

**Figure 2:**
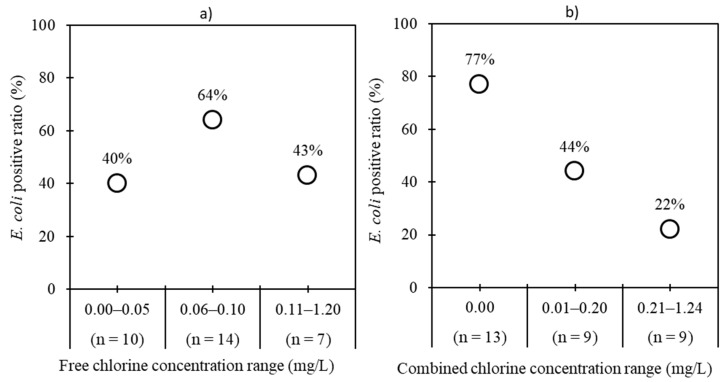
*E. coli* concentrations plotted against (**a**) free chlorine concentration categories and (**b**) combined chlorine concentration categories in tanker filling station samples.

Chlorine is a widely used disinfectant employed within water treatment procedures in the valley. We examined the relationship between the *E. coli*-positive ratio and the concentrations of free and combined chlorine within TFS samples. Figure 2 shows the positive ratios of *E. coli* in water samples from TFSs in different categories of free (Figure 2a) and combined (Figure 2b) chlorine concentrations. The positive ratios of *E. coli* gradually decreased with an increase in free and combined chlorine concentrations, except for the category of 0.00–0.05 mg/L free chlorine. The concentration of total chlorine in this category was 0.01–0.59 mg/L. When water samples were divided into three categories based on total chlorine concentration, the positive ratios of *E. coli* were 60% (6/10), 64% (7/11), and 30% (3/10) for 0.01–0.04, 0.05–0.34, and 0.35–1.42 mg/L of chlorine concentration, respectively.

### 2.2. Detection of Pathogenic Viruses

Table 2 shows the results of testing for six pathogenic viruses—AiV-1, EVs, HAdVs, NoVs-GI and GII, and RVAs—analyzed for TFS and WT samples. Of the 17 TFSs, EVs, NoVs-GII, HAdVs, and RVAs were detected at 10, five four, and two TFSs, respectively. Between two and four pathogenic viruses were detected at six TFSs. Among all the pathogenic viruses tested, EVs were the most prevalent viruses in TFS samples, with a positive ratio of 35% (11/31), followed by NoVs-GII (23%, 7/31), HAdVs (13%, 4/31), and RVAs (6%, 2/31). On the other hand, NoVs-GII were most frequently detected in WT samples (20%, 6/30), followed by EVs (13%, 4/30), RVAs (10%, 3/30), and HAdVs (7%, 2/30). The detection frequency of EVs was significantly higher in TFS samples (35%, 11/31) than in WT samples (13%, 4/30) (χ^2^-test; *p* < 0.05). However, no significant differences in the detection frequencies of NoVs-GII (χ^2^-test; *p* > 0.05), HAdVs, and RVAs (fisher exact-test; *p* > 0.05) between TFS and WT samples were observed. At least one pathogenic virus was detected in 45% (14/31) of TFS samples and 27% (8/30) of WT samples. Furthermore, NoVs-GII were detected at two TFSs continuously during both seasons. However, AiV-1 and NoVs-GI were undetected in any of the sampled TFSs and WTs.

### 2.3. Detection of Host-Associated Fecal Markers

Microbial source tracking was conducted for TFS samples using previously validated host-associated *Bacteroidales* [26], mtDNA, and viral markers. Table 3 shows the results of the detection of fecal markers in the TFS samples. The frequency of at least one human-associated marker (39%, 12/31) detection was significantly higher than ruminant-associated marker (14%, 3/22) (χ^2^-test; *p* < 0.05). Chicken- and pig-associated markers were detected in 3% (1/31) and 5% (1/22) of TFS samples, respectively. Dog-associated markers were not detected in any of the TFS samples. At least one human- and ruminant-associated markers were detected at 10 and 3 out of 17 TFSs tested, respectively. Human- and animal-mixed fecal contamination was observed at two TFSs. For one TFS, contaminations from all the tested hosts were judged, with the exception of dog. Animal-associated fecal markers were detected at three TFSs, all of which were located in the peri-urban area where agriculture and livestock farming are common. At least one pathogenic virus was detected in 69% (9/13) and 33% (6/18) of samples that tested positive and negative for fecal markers, respectively. At least one fecal marker was detected at nine (75%) out of 12 TFSs within which pathogenic viruses were detected. In addition, human-associated fecal markers were continuously detected at two TFSs during both seasons.

## 3. Discussion

Fifty-two percent (16/31) of TFS samples were contaminated with *E. coli*, indicating poor performance of the treatment plants. *E. coli* detection in 70% (21/30) of WT samples with concentrations higher than the World Health Organization (WHO) guideline values for drinking water (<1 MPN/100 mL) indicated the unsuitability of this tanker water for drinking purposes [27]. When the relationship between *E. coli* detection and free or combined chlorine concentrations was examined, there was a decreasing trend in the positive ratios of *E. coli* as the concentrations of free and combined chlorine increased. However, there was a low positive ratio of *E. coli* in the category 0.00–0.05 mg/L of free chlorine, which could be due to the presence of combined chlorine. This result suggested that chlorine application could be a useful measure for lowering the concentration of *E. coli* in WTs. Although the difference was not significant, the concentrations of *E. coli* in WT samples were higher compared with their corresponding TFS samples. *E. coli* was detected in 46% (6/13) of WT samples that were negative for the corresponding TFSs. These results indicated that tankers are not disinfected and/or cleaned regularly. A similar result was obtained in Lebanon, where eight tankers had higher concentrations of fecal coliforms than their water sources [28].

High positive ratios for the potential indicators of pathogenic viruses, PMMoV and TMV, in TFS and WT samples indicated that other water-transmitted viral pathogens, such as astroviruses and hepatitis A and E viruses, could be present, for which testing was not performed in this study. Group A rotaviruses, which are the major causative agent of gastroenteritis in Nepal [29,30,31], were detected in 10% (3/30) of WT samples. Previous studies have reported the detection of pathogenic viruses—such as AiV-1, EVs, HAdVs, NoVs-GI, NoVs-GII, and RVAs—in groundwater and river water in the valley, which are the major sources of tanker water [1,13,14,32,33]. A tap water sample supplied by a tanker in the valley was found to be contaminated with pathogens, including HAdVs and *Vibrio cholerae*, further indicating the unsuitability of tanker water for drinking purposes [11]. In addition, NoVs-GI and HAdVs were also detected in two and one samples, respectively, out of five water tankers sampled in the valley, and enteric viruses were found to be responsible for gastroenteritis in children suffering from diarrhea [33]. A previous study reported a high risk of diarrheal infections for consumers of raw vegetables washed with tanker water or other water sources in the valley [34]. High positive ratios of fecal indicator bacteria and pathogenic viruses in TFS samples show that the employed treatment systems were not sufficient to eliminate the pathogens tested. 

When the possible sources of such pathogenic viruses and fecal indicator bacteria in these water samples were analyzed by an MST technique, 39% (12/31) and 14% (3/22) of water samples were judged to be contaminated with human and ruminant feces, respectively. The detection of ruminant fecal markers has been previously reported in tanker water [11]. This could be due to the use of groundwater and surface water by the TFSs, in which human and animal fecal contaminations have been reported [11,35,36]. A previous study reported the possible transmission of enteric viruses from feces to children consuming water from sources contaminated by these viruses [33]. The detection of pathogenic viruses and fecal markers in the same sample indicated that these viruses might have originated from the feces of humans and animals. The detection of the animal fecal markers, mostly in samples originating from the peri-urban areas of the valley, could be due to the land use pattern of those areas where agriculture and farming are commonly practiced [35]. In Cambodia, animals were found to be responsible for the fecal pollution of water sources in agricultural areas [37], and livestock ownership is significantly associated with water contamination in Ghana and Bangladesh [38]. These results indicate a high risk to public health, which requires immediate action for control and prevention of possible disease outbreaks.

Groundwater, a major source for tanker water in Nepal [6,9], is contaminated by human and animal feces [26,35]. Despite an effort to ban on the implementation of deep tube wells within a 200 m distance of riverbanks, some TFSs are still found near riverbanks. Mixing of river water with nearby groundwater has been previously reported [39]. These reasons may contribute to the poor microbial quality of tanker water. This study showed that an increase in the concentrations of free and combined chlorine was associated with decreased concentrations of *E. coli* in WT samples, suggesting that chlorine application could be one of the measures used to lower the concentration of *E. coli* in WTs.

In conclusion, this study reports that the water supplied to the TFSs and WTs to the public are contaminated with fecal indicator bacteria and pathogenic viruses. This study also highlighted the use of host-associated *Bacteroidales*, mtDNA, and viral genetic markers to identify the sources of fecal pollution. The major source of microbial contamination was judged to be human feces, indicating that better infrastructure and management practices should be implemented. The increased microbial contamination present in WTs compared with that of TFS samples suggests the importance of regular cleaning and disinfection of the WTs.

## 4. Materials and Methods

### 4.1. Collection of Water Samples

Altogether, 31 TFS water samples were collected from 17 TFSs during the dry (March; n = 16) and wet (August; n = 15) seasons of 2016, and from 30 WTs during the dry (n = 17) and wet (n = 13) seasons of the same year. The water supplied by the tanker water treatment plants or TFSs to the tankers or the vehicles that carry water are referred to as TFS samples, and the water distributed by these vehicles to the public are referred to as WT samples. Water samples were collected in two 100 mL and five 1 L plastic bottles, which were washed with pure water prior to autoclaving, for each of the TFS and WT samples. Chlorine concentrations of WT samples were measured using a portable water analyzer colorimeter (HACH, Loveland, Co, USA). All samples were stored cold, transported to the laboratory, kept at 4 °C, and processed within 4 h.

### 4.2. Detection of Total Coliforms and E. coli

Total coliforms and *E. coli* were determined by the MPN method using a Colilert reagent (IDEXX Laboratories, Westbrook, CA, USA), as described previously [14,40].

### 4.3. Concentration and Extraction of Bacterial, mtDNA, and Viral Markers and Viruses

Bacterial and mtDNA were extracted using a CicaGeneus DNA Extraction Reagent (Kanto Chemical, Tokyo, Japan), as previously described [26,35]. Briefly, 100 mL of a water sample was filtered using a disposable filter unit preset with a nitrocellulose membrane (diameter, 47 mm; pore size, 0.22 µm; Nalgene, Tokyo, Japan). The filter membrane was transferred into a 50 mL tube and 5 mL of Tris–EDTA buffer (pH 7.4) was added. The resuspended sample was processed after repeated shaking and mixing by vortexing. A final volume of 300 μL of DNA extract was obtained by processing 160 µL of the resuspended sample with 20 µL of Buffer A and 200 µL of Buffer B.

An electronegative membrane-vortex method [41] was used as described previously with some modifications for virus concentration of the water samples [13,14,36]. Briefly, for the concentration step, 50 mL of 2.5 mol/L MgCl_2_ was added to the 5 L water sample and filtered using a mixed cellulose-ester membrane (pore size, 0.8 µm; diameter, 90 mm; Merck Millipore, Billerica, MA, USA). Filter membrane was removed from the filter holder and vigorous vortexing of the membrane was performed with elution buffer in a 50 mL plastic tube to recover an eluate (~15 mL), as mentioned previously [13,14]. Subsequently, the eluate was centrifuged at 2000 × *g* for 10 min at 4 °C, followed by filtration of supernatant using a disposable membrane filter unit (pore size, 0.45 µm; diameter, 25 mm; Advantec, Tokyo, Japan). Finally, the filtrate was further concentrated using a Centriprep YM-50 ultrafiltration device (Merck Millipore) to obtain a virus concentrate, following the manufacturer’s protocol. Viral DNA was extracted using a QIAamp DNA Mini Kit (QIAGEN, Hilden, Germany) from 200 µL of viral concentrate to obtain 200 µL of DNA extract. Similarly, a QIAamp Viral RNA Mini Kit (QIAGEN) was used to obtain a 60 µL RNA extract from 140 µL of viral concentrate, following the manufacturer’s protocol. Both DNA and RNA extractions were performed using a QIAcube automated platform (QIAGEN). Thirty microliters of viral RNA was subjected to reverse transcription using a High-Capacity cDNA Reverse Transcription Kit (Applied Biosystems, Foster City, CA, USA) to obtain 60 µL of cDNA.

### 4.4. Detection of Viruses and Fecal Markers

The effect of qPCR inhibition was evaluated in this study as recommended elsewhere [42]. Porcine teschovirus (PoTeVs), as a control, was inoculated into DNA extract and recovered by qPCR. For quantitative PCR (qPCR), 2.5 µL of template DNA/cDNA was added to a mixture of 22.5 µL containing 12.5 µL Probe qPCR Mix (Takara Bio, Kusatsu, Japan), 7.0 µL PCR-grade water, 1.0 µL each of 10 pmol/µL forward and reverse primers, and 1.0 µL of the 5 pmol/µL TaqMan (MGB) probe. Table 4 shows the sequences of primers and probes used in this study. For the quantification of genomes, a Thermal Cycler Dice Real Time System TP800 (Takara Bio) was used. The thermal cycle conditions for all the tested assays (BacHum [17], BacR [18], Pig2Bac [19], Bovine- and Swine-mtDNA [20], Dog-mtDNA [21], AiV-1 [43], BKPyVs and JCPyVs [44], ChkPVs [24], and PoAdVs [25]) were as follows: 95 °C for 30 s, followed by 45 cycles at 95 °C for 5 s, and 60 °C for 30 s, except for EVs [45,46], PMMoV [47,48], RVAs [49], and TMV [50] (60 °C for 60 s), HAdVs [51], NoVs-GI, and NoVs-GII [52] (58 °C for 30 s), and PoTeVs [53] (56 °C for 30 s). For the determination of the genome copy number of each virus, a standard curve was plotted using six 10-fold serial dilutions of artificially synthesized plasmid DNA containing the amplification region. The amplification efficiencies of standard curves ranged from 78% to 123%. The calculated mean efficiency of process control was 141 ± 32% (n = 30), suggesting that there was no inhibition during qPCR.

In all qPCR runs, unknown and standard samples and negative controls were run in duplicate. A negative control was included in every run. The sample was judged positive if the respective marker was detected in at least one of the two wells with the threshold cycle value of ≤40.

### 4.5. Statistical Analysis

An independent *t*-test was used for the comparison of the *E. coli* concentrations between WT and TFS samples and for comparing the concentrations of *E. coli* in WT samples between dry and wet seasons. In addition, a paired *t*-test was used to compare the concentrations of *E. coli* between WT and corresponding TFS water samples. The detection frequencies of pathogenic viruses in TFS and WT samples were compared using χ^2^ and Fisher Exact tests. Similarly, the χ^2^ test was used for the comparison of the detection frequencies of human- and ruminant-associated markers in TFS samples. For negative samples, the one-tenth value of the limit of detection (1 MPN/100 mL for *E. coli*) was used. For statistical analyses, SPSS version 23 (IBM Corporation, Armonk, USA) was used, and values were considered significant at *p* < 0.05.

## Figures and Tables

**Table 1:** Positive ratios and concentrations of fecal indicator bacteria and index viruses in tanker filling station and water tanker samples.

Water Sample	No. of Tested Samples	Fecal Indicator Bacteria				Index Viruses			
		*E. coli*		Total Coliforms		PMMoV		TMV	
		No. of Positive Samples (%)	Concentration ^a^ (log MPN ^b^/100 mL)	No. of Positive Samples (%)	Concentration ^a^ (log MPN ^b^/100 mL)	No. of Positive Samples (%)	Concentration ^a^ (log copies/L)	No. of Positive Samples (%)	Concentration ^a^ (log copies/L)
Tanker filling station	31	16 (52)	0.0–4.0	27 (87)	0.0–5.4	22 (71)	1.7–4.7	28 (90)	2.7–6.0
Water tanker	30	21 (70)	0.0–3.5	27 (90)	1.0–4.8	22 (73)	2.1–4.9	29 (97)	2.8–6.3
Total	61	37 (61)		54 (89)		44 (72)		57 (93)	

^a^ Range of concentrations among positive samples. ^b^ MPN, most probable number.

**Table 2:** Positive ratios and concentrations of pathogenic viruses in tanker filling station and water tanker samples.

Water Sample	No. of Tested Samples	AiV-1		EVs		HAdVs		NoVs-GI		NoVs-GII		RVAs		At Least One Pathogen Detected
		No. of Positive Samples (%)	Conc. ^a^ (log copies/L)	No. of Positive Samples (%)	Conc. ^a^ (log copies/L)	No. of Positive Samples (%)	Conc. ^a^ (log copies/L)	No. of Positive Samples (%)	Conc. ^a^ (log copies/L)	No. of Positive Samples (%)	Conc. ^a^ (log copies/L)	No. of Positive Samples (%)	Conc. ^a^ (log copies/L)	No. of Positive Samples (%)
Tanker filling station	31	0 (0)	NA	11 (35)	2.7–4.6	4 (13)	3.6–4.9	0 (0)	NA	7 (23)	2.0–3.9	2 (6)	3.3–3.7	14 (45)
Water tanker	30	0 (0)	NA	4 (13)	3.1–4.6	2 (7)	4.3–5.0	0 (0)	NA	6 (20)	1.8–4.5	3 (10)	2.8–3.4	8 (27)
Total	61	0 (0)		15 (25)		6 (10)		0 (0)		13 (21)		5 (8)		22 (36)

^a^ Range of concentrations among positive samples; NA, not applicable.

**Table 3:** Detection of fecal-source markers in tanker filling station samples.

Fecal Markers		Detection % (No. of Positive Samples/No. of Tested Samples)	Concentration ^d^ (log copies/L)
Human-	BacHum ^a^	5 (1/22)	6.3
	HAdVs ^b^	13 (4/31)	3.6–4.9
	BKPyVs ^b^	29 (9/31)	4.9–5.7
	JCPyVs ^b^	10 (3/31)	5.0–5.9
	At least one human marker	39 (12/31)	3.6–6.3
Ruminant-	BacR ^a^	14 (3/22)	5.4–5.9
	Bovine mtDNA^c^	0 (0/22)	NA^e^
Pig-	Pig2Bac ^a^	5 (1/22)	6.1
	PoAdVs ^b^	0 (0/31)	NA
	Swine mtDNA ^c^	0 (0/22)	NA
Dog-	Dog mtDNA ^c^	0 (0/22)	NA
Chicken-	ChkPVs ^b^	3 (1/31)	3.4

^a^*Bacteroidales* marker; ^b^ Viral marker; ^c^ Mitochondrial DNA marker; ^d^ Range of concentrations among positive samples; ^e^ NA, not applicable.

**Table 4:** Primer and probe sequences used in this study.

Assay	Primer/Probe	Sequence (5′–3′)	Product Length (bp)	Reference
AiV-1	Forward primer	GTCTCCACHGACACYAAYTGGAC	108–111	[43]
	Reverse primer	GTTGTACATRGCAGCCCAGG		
	TaqMan MGB probe	FAM-TTYTCCTTYGTGCGTGC-MGB-NFQ		
BacHum	Forward primer	TGAGTTCACATGTCCGCATGA	82	[17]
	Reverse primer	CGTTACCCCGCCTACTATCTAATG		
	TaqMan probe	FAM-TCCGGTAGACGATGGGGATGCGTT-TAMRA		
BacR	Forward primer	GCGTATCCAACCTTCCCG	118	[18]
	Reverse primer	CATCCCCATCCGTTACCG		
	TaqMan MGB probe	FAM-CTTCCGAAAGGGAGATT-MGB-NFQ		
BKPyVs	Forward primer	GGCTGAAGTATCTGAGACTTGGG	78	[44]
	Reverse primer	GAAACTGAAGACTCTGGACATGGA		
	TaqMan probe	FAM-CAAGCACTGAATCCCAATCACAATGCTC-TAMRA		
Bovine-mtDNA	Forward primer	CAGCAGCCCTACAAGCAATGT	191	[20]
	Reverse primer	GAGGCCAAATTGGGCGGATTAT		
	TaqMan probe	FAM-CATCGGCGACATTGGTTTCATTTTAG-TAMRA		
ChkPVs	Forward primer	AGTCCACGAGATTGGCAACA	82	[24]
	Reverse primer	GCAGGTTAAAGATTTTCACG		
	TaqMan probe	FAM-AATTATTCGAGATGGCGCCCACG-TAMRA		
Dog-mtDNA	Forward primer	GGCATGCCTTTCCTTACAGGATTC	109	[21]
	Reverse primer	GGGATGTGGCAACGAGTGTAATTATG		
	TaqMan probe	FAM-TCATCGAGTCCGCTAACACGTCGAAT-TAMRA		
EVs	Forward primer	CCTCCGGCCCCTGAATG	195	[45]
	Reverse primer	ACCGGATGGCCAATCCAA		
	TaqMan probe	FAM-CCGACTACTTTGGGTGTCCGTGTTTC-TAMRA		[46]
HAdVs	Forward primer	GCCACGGTGGGGTTTCTAAACTT	132	[51]
	Reverse primer	GCCCCAGTGGTCTTACATGCACATC		
	TaqMan probe	FAM-TGCACCAGACCCGGGCTCAGGTACTCCGA-TAMRA		
JCPyVs	Forward primer	GGAAAGTCTTTAGGGTCTTCTACCTTT	89	[44]
	Reverse primer	ATGTTTGCCAGTGATGATGAAAA		
	TaqMan probe	FAM-GATCCCAACACTCTACCCCACCTAAAAAGA-TAMRA		
NoVs-GI	Forward primer	CGYTGGATGCGNTTYCATGA	85	[52]
	Reverse primer	CTTAGACGCCATCATCATTYAC		
	TaqMan probe	FAM-AGATYGCGATCYCCTGTCCA-TAMRA		
NoVs-GII	Forward primer	CARGARBCNATGTTYAGRTGGATGAG	98	[52]
	Reverse primer	TCGACGCCATCTTCATTCACA		
	TaqMan probe	FAM-TGGGAGGGCGATCGCAATCT-TAMRA		
Pig2Bac	Forward primer	GCATGAATTTAGCTTGCTAAATTTGAT	117	[19]
	Reverse primer	ACCTCATACGGTATTAATCCGC		
	TaqMan MGB probe	FAM-TCCACGGGATAGCC-MGB-NFQ		
PMMoV	Forward primer	GAGTGGTTTGACCTTAACGTTTGA	68	[47]
	Reverse primer	TTGTCGGTTGCAATGCAAGT		[48]
	TaqMan MGB probe	FAM-CCTACCGAAGCAAATG-MGB-NFQ		[47]
PoAdVs	Forward primer	AACGGCCGCTACTGCAAG	68	[25]
	Reverse primer	AGCAGCAGGCTCTTGAGG		
	TaqMan MGB probe	FAM-CACATCCAGGTGCCGC-MGB-NFQ		
PoTeVs	Forward primer	CACCAGCGTGGAGTTCCTGTA	66	[53]
	Reverse primer	AGCCGCGACCCTGTCA		
	TaqMan probe	FAM-TGCAGGACTGGACTTG-TAMRA		
RVAs	Forward primer	CAGTGGTTGATGCTCAAGATGGA	131	[49]
	Reverse primer	TCATTGTAATCATATTGAATACCA		
	TaqMan probe	FAM-ACAACTGCAGCTTCAAAAGAAGWGT-TAMRA		
Swine-mtDNA	Forward primer	ACAGCTGCACTACAAGCAATGC	197	[20]
	Reverse primer	GGATGTAGTCCGAATTGAGCTGATTAT		
	TaqMan probe	FAM-CATCGGAGACATTGGATTTGTCCTAT-TAMRA		
TMV	Forward primer	CAAGCTGGAACTGTCGTTCA	120	[50]
	Reverse primer	CGGGTCTAAYACCGCATTGT		
	TaqMan probe	FAM-CAGTGAGGTGTGGAAACCTTCACCACA-TAMRA		
FAM, 6-carboxyfluorescein; MGB, minor groove binder; NFQ, nonfluorescent quencher; TAMRA, 5-carboxytetramethylrhodamine.				
